# Editorial: Molecular Basis of the Response of Photosynthetic Apparatus to Light and Temperature Stress

**DOI:** 10.3389/fpls.2017.00288

**Published:** 2017-03-06

**Authors:** Rafael Picorel, Miguel Alfonso, Maya Velitchkova

**Affiliations:** ^1^Estación Experimental de Aula Dei-CSICZaragoza, Spain; ^2^Department of Photoexcitable Membranes, Institute of Biophysics and Biomedical Engineering-BASSofia, Bulgaria

**Keywords:** photosynthesis, abiotic stresses, plant productivity, instrumentation, plant protection

The fast growing world population requires an increase in plant productivity. With the natural biodiversity and the present available technologies, it would be difficult to cope with this huge task. Plants are sessile organisms and are subjected to continuous environmental constrains as climate change, pollution, soil degradation, and so forth due to natural events and anthropogenic activities. Abiotic stress is responsible for plant productivity losses, including crops, grass lands and forests. Thus, reducing plant stress pressure would eventually contribute to attain future food and bioenergy demand, and protect the environment. As a constant exposure to suboptimal growth conditions, plants have developed numerous strategies to mitigate or eliminate stress effects.

Photosynthesis is an intricate and crucial function in plant productivity, and the ability of plants to adapt to changing environments is related to the plasticity of photosynthesis. Some plant species are more tolerant than others to environmental constraints. Natural biodiversity offers, therefore, some clues to exploit that plasticity that can be evaluated using available instrumentation. The obtained information could be transferred to more interested plants for food or bioenergy supply. This Special Issue covers certain aspects of the effects and protections of the photosynthetic machinery under some abiotic stresses.

Abiotic stress significantly reduces plant productivity and damages plant ecosystems. Roncel et al. report the negative effects of nutrition deficiency on photosynthesis. Drought, salinity, nutrition, high-light, UV-radiation, increasing concentration of atmospheric CO_2_ and CH_4_, global warming, pollution, heavy metals, and so forth are frequent environmental stresses that affect photosynthesis and plant productivity. Plants have developed protective mechanisms that can mitigate or eliminate the negative effects on photosynthesis. As an example Yamamoto describes how membrane fluidity can modulate the effects of heat and light stress. Some mechanisms are expressed in most stress conditions and most plants but others are stress and plant specific. Most of stress effects can be partially or totally reversible provided the damage was not too severe. This is why is so important the development of non-invasive early detection techniques to avoid the progression of damages. The measurements can be made at small scale but also in larger plant populations and ecosystems. In that sense, imaging analysis and 3D reconstruction supported by remote sensing detection are becoming strong tools to better understand plant physiology and development under changing environmental conditions.

Most environmental stresses affect photosynthesis. Measuring photosynthetic parameters is the easier and faster way to assess the type and degree of stressful conditions. Some stresses as drought produces a rapid hormone signals that affects gas exchange. Other environmental stresses (high light, UV-radiation, nutrition, drought, salinity, heat) induce remarkable changes in pigments, lipids, proteins and thylakoid structure. Some of them could appear simultaneously making synergic harmful effects. PSII is normally the most affected under abiotic stress, frequently reducing chlorophyll (Chl) content, especially Chl *b*, giving rise to measurable chlorosis. For instance Pospíšil described the production of ROS by the PSII under light and temperature stress. In high-light, PSII is affected by a process called photoinhibition. The changes in chemical composition and reorganization of thylakoids normally hamper excitonic energy transfer and electron transfer rate. Most of abiotic stresses induce pigment, lipid, and protein changes, which frequently produce reorganization of thylakoids. The article of Ware et al. sheds light on some aspects of the carotenoids photoprotection.

Plants have developed protective mechanisms to maintain photosynthesis efficiency. Beside thylakoids and protein-complex reorganization, under abiotic stress a cascade of cell signaling takes place to induce synthesis of transcription factors, ROS molecules, phytohormones, solutes, kinases, phosphatases and proteases to protect photosynthesis. One of the most studied protective mechanism corresponds to the non-photochemical quenching (NPQ) induced by the xanthophyll cycle (VAZ cycle) in high-light to dissipate excess excitation energy as heat. Plants with more active VAZ cycle are more tolerant to high-light and other environmental stresses. An extensive search for this natural diversity would be an important target in plant sciences. This natural plasticity could then be transferred to plants with more added values.

Chlorophylls are molecules with strong absorbance and highly fluorescent, and their content and distribution within thylakoids are sensitive to most abiotic stresses. This makes Chls very useful sensing probe to study plant stress. Some techniques that use Chl properties are the following: (a) Fluorescence induction and decay to calculate important photosynthetic parameters as Fv/Fm, Y′ (II), ΔF/Fm′, OIJP, PSMT, NPQ, etc.; (b) Absorption techniques to measure the greenness and thickness of leaves giving CCI and SPAD indexes; (c) Remote sensing to detect signals at variable distances from the target, to study plants, cultivars, forests and ecosystems.

Plant productivity increase is a must in a growing population. Despite the recent encouraging news about the reduction of the ozone layer hole and the increase of greenness in certain latitudes that can compensate by about half the tropical deforestation, environmental pressure will continue in the future. Thus, obtaining more tolerant natural or transgenic plants along with the use of appropriate measurements should be a priority for plant scientists. The precise phenotyping of traits (phenomes) caused by abiotic stress, introduction of a foreign gene, plant acclimation and adaptation, and so forth is at present a bottleneck in plant sciences. We have to fulfill the gap between genotyping and phenotyping. Since the phenotype can be defined as the result of genotype × environment, the application of phenomics is suitable to identify photosynthetic traits under stress. Phenomics should be done at all levels of complexity from roots, leaves, plants, cultivars or forests in controlled environments and in the field. High-throughput phenotyping tools will offer invaluable possibilities to monitor precisely the appearance and development of traits under environmental stress, facilitating the selection of natural or genetically modified plants more tolerant to stress. To that end, multiple remote sensing techniques, robotics, imaging analysis, data management and 3D reconstructions can be used to assess the status and development of plants, cultivars or larger ecosystems under abiotic stress. Several of these phenomics techniques take advantage of the unique properties of the photosynthetic apparatus.

## Author contributions

RP wrote the Editorial, MV and MA contributed in designing the main body of the article, and participated with improving suggestions, and the acceptance of the final version of the Editorial.

## Funding

Work in the laboratory of RP and MA was supported by Grant AGL2014-55300-R from Ministry of Economy and Competitiveness (MINECO) of Spain. Work in MV laboratory was supported by Swiss National Science Foundation under BSRP (IZEBZO-143169/1).

### Conflict of interest statement

The authors declare that the research was conducted in the absence of any commercial or financial relationships that could be construed as a potential conflict of interest.

